# Dieting reverses histone methylation and hypothalamic AgRP regulation in obese rats

**DOI:** 10.3389/fendo.2023.1121829

**Published:** 2023-02-01

**Authors:** Kayla Rapps, Tatiana Kisliouk, Asaf Marco, Aron Weller, Noam Meiri

**Affiliations:** ^1^ Faculty of Life Sciences, Bar Ilan University, Ramat-Gan, Israel; ^2^ Institute of Animal Science, Agricultural Research Organization, The Volcani Center, Rishon LeZiyyon, Israel; ^3^ Gonda Multidisciplinary Brain Research Center, Bar Ilan University, Ramat-Gan, Israel; ^4^ Neuro-Epigenetics Laboratory, Faculty of Agriculture, Food and Environment, The Hebrew University of Jerusalem, Rehovot, Israel; ^5^ Department of Psychology, Bar Ilan University, Ramat-Gan, Israel

**Keywords:** obesity, caloric restriction, histone modification, epigenetics, hypothalamus

## Abstract

**Introduction:**

Although dieting is a key factor in improving physiological functions associated with obesity, the role by which histone methylation modulates satiety/hunger regulation of the hypothalamus through weight loss remains largely elusive. Canonically, H3K9me2 is a transcriptional repressive post-translational epigenetic modification that is involved in obesity, however, its role in the hypothalamic arcuate nucleus (ARC) has not been thoroughly explored. Here we explore the role that KDM4D, a specific demethylase of residue H3K9, plays in energy balance by directly modulating the expression of AgRP, a key neuropeptide that regulates hunger response.

**Methods:**

We used a rodent model of diet-induced obesity (DIO) to assess whether histone methylation malprogramming impairs energy balance control and how caloric restriction may reverse this phenotype. Using ChIP-qPCR, we assessed the repressive modification of H3K9me2 at the site of AgRP. To elucidate the functional role of KDM4D in reversing obesity via dieting, a pharmacological agent, JIB-04 was used to inhibit the action of KDM4D *in vivo*.

**Results:**

In DIO, downregulation of *Kdm4d* mRNA results in both enrichment of H3K9me2 on the AgRP promoter and transcriptional repression of AgRP. Because epigenetic modifications are dynamic, it is possible for some of these modifications to be reversed when external cues are altered. The reversal phenomenon was observed in calorically restricted rats, in which upregulation of *Kdm4d* mRNA resulted in demethylation of H3K9 on the *AgRP* promoter and transcriptional increase of AgRP. In order to verify that KDM4D is necessary to reverse obesity by dieting, we demonstrated that *in vivo* inhibition of KDM4D activity by pharmacological agent JIB-04 in naïve rats resulted in transcriptional repression of AgRP, decreasing orexigenic signaling, thus inhibiting hunger.

**Discussion:**

We propose that the action of KDM4D through the demethylation of H3K9 is critical in maintaining a stable epigenetic landscape of the AgRP promoter, and may offer a target to develop new treatments for obesity.

## 1 Introduction

Obesity is a complex disease that is prevalent worldwide (1–3) and is associated with adverse health outcomes and risk factors (4–12). To improve global health and attenuate metabolic disease, it is imperative to better understand the molecular mechanisms modulating weight loss. The process of reversing obesity and rebalancing the metabolic and endocrine equilibrium through dieting is not fully understood (13). There is developing evidence that epigenetic targets may be central in promoting weight loss (14–16).

Extreme changes in body weight drive epigenetic modifications both systemically (16–21) and specifically in the hypothalamus (22–26). The hypothalamic arcuate nucleus (ARC) is the homeostatic energy center of the brain, integrating the input from peripheral hormones, i.e., leptin, ghrelin, and insulin, to regulate satiety and hunger signals through counter-expression of anorexigenic, i.e., CART (cocaine- and amphetamine-regulated transcript)/POMC (pro-opiomelanocortin) and orexigenic, i.e., NPY (neuropeptide Y)/AGRP (agouti-related protein) neuropeptide signaling. A delicate balance in signaling maintains a steady body weight set-point through circumscribing hunger, satiation, and energy output (22, 27–30). Aberrant expression of these neuropeptides in the ARC partially mediates dysregulated feeding patterns in diet-induced obese (DIO) rodents (27, 29, 31, 32). AgRP has been specifically implicated in mediating feeding and energy balance in animals. As an orexigenic neuropeptide released by the ARC, AgRP partially mediates feeding and energy balance. *AgRP* expression is reduced when animals are satiated, leading to a reduced drive to feed. Activation of AgRP leads to the development of obesity, not only through hyperphagia, but also *via* reduction in voluntary exercise (33–36). Knockout or ablation of AgRP lead to uncontrolled anorexia, together with loss in weight and adipose tissue (33, 37, 38).

It has been shown that hypothalamic dysfunction could be partially reversed by weight-loss *via* alterations in epigenetic markings on relevant genes or chromatin (14, 39, 40), for example, demethylation of the *Pomc* promoter after body weight decrease (29), or restoration of baseline methylation patterns on the *Lepr* promoter in dieting obese rats (41).

Lysine 9 di-and tri-methylation on histone 3 (H3K9me2 and H3K9me3) are hallmarks of transcriptional repression (42) and recent evidence shows that changes in enrichment of H3K9me2/3 are prominent in obesity (43–48). Obese rodent models have been found to have enhanced enrichment of H3K9me2/3 in adipose tissue specifically in genes related to inflammation, lipogenesis and energy metabolism (44–46). Further, a systemic absence of normal H3K9 methylation patterns resulted in decreased energy expenditure, reduced oxygen consumption and impaired adaptive thermogenesis in rats (46). Importantly, recent studies have shown that H3K9 methylation in various brain regions modulates obesity (47, 49).

The maintenance of chromatin architecture by histone-tail methylation regulation at lysine residues is achieved by the dynamic coordination of methyl transferases (KMTs) and demethylases (KDMs) (50, 51). KDM4D is an enzyme that specifically demethylates the H3K9 residue (45, 52, 53) and is conventionally involved in DNA damage repair (54) and DNA replication (53). The novel potential role of KDM4D in the onset and reversal of obesity has not yet been elucidated. Here we used a low dose of JIB-04, which has not yet been used in the context of weight control, to pharmacologically inhibit the action of KDM4D in naïve rats. JIB-04 is a pan-selective inhibitor of various proteins in the KDM family, with a high selectivity for KDM4D (55) and has been used both *in vitro* to inhibit cancer cell activity and *in vivo* to inhibit different types of cancer growths (55–57) and it increases survival rates in mice (55). Unlike other KDM inhibitors, JIB-04 is the only known agent to function *in vivo* and successfully pass the blood-brain barrier (57).

In this research, we were interested in uncovering the molecular mechanism by which KDM4D modulates the expression of genes in the ARC after diet-induced obesity, and specifically focus on the reversal of this hypothalamic dysfunction through caloric restriction.

## 2 Materials and methods

### 2.1 Animals

Wistar rats were bred at Envigo RMS (Jerusalem, Israel) and were housed at Bar-Ilan University’s rodent facility from standard weaning age (PND 21) onward. Rat diet was either standard chow (2018SCF; Teklad Global 6% Fat Rodent Diet; Harlan, Madison, WI, USA) or 60% high-fat diet (D12492; Research Diets, Inc., New Brunswick, NJ, USA) as indicated in experimental timelines. Rats were given free access to water throughout the entire study. Room temperature was maintained at 22 ± 2°C, with a standard 12-hour lights on/off schedule (lights on at 07:00 hr). Rats were housed in pairs, except for during Phase II of experiment 1, and for 24 hours post JIB-04 administration during food intake assessment in experiment 2. All experimental procedures were approved by the Bar-Ilan University Animals Care and Use Committee and were performed in accordance with the American Psychological Association and Society for Neuroscience guidelines. All efforts were made to minimize suffering and the number of rats used.

### 2.2 Experimental outline

#### 2.2.1 Experiment 1: Caloric restriction after diet-induced obesity

**Figure 1:**
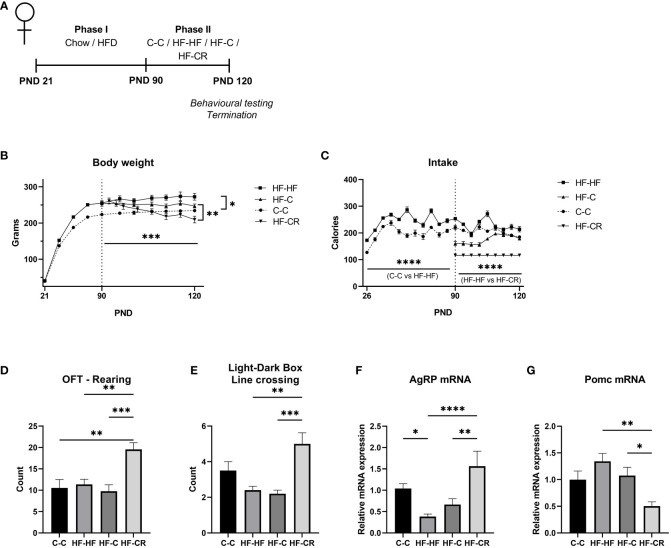
Caloric restriction results in reduction of body weight, increased explorative behaviours and changes in classic-energy balanced gene expression in the ARC of diet-induced obese female rats. **(A)** Experimental timeline. In Phase I (PND 21-90), rats were raised on either chow (n= 24) or HFD (n= 72). In Phase II (PND 90-120) rats were assigned to various treatment groups (n=24), with body weight normalized between the HFD groups. C-C (Chow-Chow) group maintained an *ad libitum* chow diet, HF-HF (HFD-HFD) group maintained an *ad libitum* HF diet, HF-C (HF-Chow) group switched from *ad libitum* chow to HFD and HF-CR (HFD-Caloric Restriction) group switched diet from *ad libitum* HFD to a 60%-calorically restricted diet of chow. At PND 120, rats were subjected to the Open Field Test and the Light-Dark Box test before termination of experiment. **(B)** The average body weight (grams) throughout the duration of the experiment. In Phase I, rats were weighed every 5 days and weighed daily in Phase II **(C)** Average caloric intake (kCal) throughout the duration of the experiment, measured by the weight difference of food remaining in the cages over 5 days during Phase I and daily in Phase II (Chow: 1 g = 3.1 kCal; HFD: 1 g = 5.24 kCal). **(D)** At the end of Phase II, rats were exposed to the Open Field test (OFT). The parameter of *rearing* counts the discrete occurrences in which the rat stands on hind legs during the test. **(E)** The rats were then subjected to the Light-Dark Box test (LDB). The parameter of *line* crossing counts the discrete occurrences in which the rat crossed between the light and dark chambers. Gene expression of the ARC was measured using RT-qPCR with primers designed for **(F)** AgRP and **(G)** Pomc. *Hprt* expression was used as the standard gene to normalize all results. Relative gene expression in the C-C group was set to 1. Data are presented as mean ± SEM, and significant effects between groups are indicated as * 0.01 < P < 0.05, **0.001 < P < 0.01, *** P < 0.001. n = 24 per group for BW and intake, n =12 per group for OFT and LDB. **** P < 0.0001.

In Phase I (PND 21-90) of the experiment (Fig 1A), the rats were raised on chow (males: n=16; females n=24) or HFD (males: n=32; females: n= 72). In Phase II (PND 90-120), rats were divided into the following four groups: (i) C-C (chow-chow) group (males: n=16; females: n=24), rats maintained an *ad libitum* chow diet; (ii) HF-HF (HFD-HFD) group (males: n=10; females: n=24), rats maintained an *ad libitum* HF diet; (iii) HF-C (HF-Chow) group (males: n=11; females: n=24), rats switched from *ad libitum* HF to *ad libitum* chow; and (iv) HF-CR (high fat diet-caloric restriction) group (males: n=11; females: n=24), rats switched from *ad libitum* HF to a 40%-calorically restricted –diet in males, and a 60%-calorically restricted diet of chow in females (58–60).

At the end of Phase II, the female rats were subjected to the Open Field test (OFT) and the Light-Dark Box Test (LDB) (61) then sacrificed. Body weight and food intake were measured every 5 days during Phase I of the experiment. In Phase II, body weight and food intake were measured daily. Caloric intake was calculated by multiplying the average amount of grams consumed per cage by 3.1 kcal for chow or 5.24 kcal for HFD. The caloric restriction was calculated based on the average intake of the HF-HF treatment group and allotted daily during the morning hours.

#### 2.2.2 Experiment 2: KDM4D inhibition

In this pharmacological experiment (**Figure 3A**), naïve rats underwent a handing period to minimize stress. Baseline feeding behaviour over a 24-hour period was assessed before any pharmacological intervention. Each rat was administered 20 mg/kg of JIB-04 (MedChemExpress; Monmouth Junction, NJ 08852, USA) or vehicle solution (10% DMSO, 90% sesame oil) intraperitoneally every-other day for a total of three treatments. Food intake was measured 24-hours following each injection. After the first injection, feeding was also assessed 6-hours post injection. Feeding patterns of each rat were normalized to their individual baseline feeding patterns that were assessed one week prior to the first drug administration.

Drug solutions were prepared fresh before each injection. 24-hours after the final drug administration, rats were subjected to the Open Field test and then sacrificed.

### 2.3 Behavioural testing

#### 2.3.1 Open field test

The open field test (OFT) was performed in a behavioural-testing room equipped with a camera and EthoVision XT (version 15) analysis software. Rats were tested individually for 5-minutes in a 1 meter-squared arena. Rats were placed in the center and were free to explore. The arena was disinfected with 96% ethanol between tests.

#### 2.3.2 Light dark box test

The light dark box (LDB) test was performed in the behavioural-testing room equipped with a camera and EthoVision XT (version 15) analysis software. The arena was comprised of an open/illuminated chamber and a covered/dark chamber, with a small opening (5 cm x 5 cm) to allow the free crossing between chambers. Rats were placed in the illuminated chamber side and were free to explore for 5-minutes. The arena was disinfected with 96% ethanol between tests.

### 2.4 Tissue collection

#### 2.4.1 Sacrifice

At the end of each experiment, rats were sacrificed *via* rapid decapitation after brief CO_2_ exposure. Brains were removed using surgical instruments and immediately frozen on dry ice and stored at -80°C.

#### 2.4.2 Neural tissue extraction

Coronal brain sections of the hypothalamus were sliced using a cryostat (-2.3 to -4.5 mm Bregma, using Paxison and Watson coordinates) and the ARC was extracted with a 1.5 mm disposable Miltex biopsy punch plunger (Bar Noar Ltd). Punches from each hemisphere were immersed in RNA Save (Biological Industries, Kibbutz Beit-Haemek, Israel) for RNA extraction, or frozen on dry ice for chromatin immunoprecipitation.

### 2.5 RNA extraction and RT-qPCR

RNA was extracted from the ARC that was stored in RNA Save. Total RNA was isolated using TriReagent (Molecular Research Center, Cincinnati, OH) according to the manufacturer’s instructions. ARC RNA was reverse-transcribed to single-stranded cDNA by Super Script II Reverse Transcriptase and oligo (dT) and random primers (Thermo Fisher Scientific, Waltham, MA, United States). Quantitative real-time PCR (qPCR) was performed with 10 ng cDNA in a StepOnePlus Real Time PCR System (Applied Biosystems) with PerfeCta SYBR Green FastMix ROX (Quanta BioSciences, Gaithersburg, MD, United States). Dissociation curves were analyzed following each qPCR to confirm the presence of only one product and the absence of primer dimer formation. The threshold cycle number (Ct) for each tested gene (X) was used to quantify the relative abundance of that gene using the formula 2^(^ *^Ct gene X-Ct Hprt^* ^)^. *Hprt* was used as the standard for mRNA expression. The primers used for qPCR were as follows: *Hprt*: F- GCGAAAGTGGAAAAGCCAAGT, R- GCCACATCAACAGGACTCTTGTAG; *Kdm4d*: F- CAACTCCCCTGCAGCAAGTAG, R- GTGCCGGTACTGCCCAACT; *AgRP*: F - AAGCTTTGGCAGAGGTGCTA, R- GACTCGTGCAGCCTTACACA); *Pomc:* F - GCTACGGCGGCTTCATGA, R- CCTCACTGGCCCTTCTTGTG.

### 2.6 ChIP assay

ChIP assays were performed as previously described (62). Briefly, frozen ARC punches were sonicated (9 rounds X 10 secs) in cell lysis buffer after 10-minute formaldehyde cross-linking. Sheared chromatin fragments (200–1000 bp) were incubated in ChIP dilution buffer with anti-H3K9me2 or anti-H3K27me2 (3 μg/sample; Cell signaling, Temecula, CA, USA). Normal mouse IgG (1 µg/sample; Cell Signaling) was used for mock immunoprecipitation (background). DNA was isolated from each immunoprecipitate and subjected to qPCR using the following primers (5′→3′): AgRP: F- aggaagtagtcacgtgtggg, R- ggacacagctcagcaacatT, AgRP (+2000 base pair downstream): F- CCTAGGTCAGTTGAGTGGCA, R- GCCACTTCTTGCTTTCCCAA). Results were normalized to input samples that were not precipitated.

### 2.7 Statistical analysis

Data were analyzed using GraphPad Prism 8 software (San Diego, CA, United States). t-tests for independent samples were used to compare between groups in experiments with two treatments and one-way ANOVA for multiple comparisons in experiments with four treatments. Two-way repeated measures ANOVA was used to analyze body weight and intake between treatment groups over time. Tukey’s multiple comparison test was used to reveal treatment differences. Data are presented as means ± standard error of the mean (SEM). In the text, statistical values (*t* and *F*) and their significance (*p*) are reported, as well as *post-hoc* multiple comparison, where appropriate. The symbols in the figures indicate significance between groups, either by t-test or *post-hoc* (# *p* < 0.1, *p < 0.05, ** *p* < 0.01, *** *p* < 0.001).

## 3 Results

### 3.1 Caloric restriction reduces body weight, increases explorative behaviours and alters expression of classic hypothalamic energy-balance genes in DIO-female rats

Given that differences in post translational histone methylation of specific residues along energy-balance related genes in the ARC nucleus moderates obesity, we were interested in trying to understand the mechanism by which KDM4D acts in weight reduced rats of an obese model. To do so, we used a rat model of diet-induced obesity followed by a period of caloric restriction.

In Phase I of the experiment, female rats were raised on either chow or HFD to cause diet- induced obesity (DIO) (**Figure 1A**). During this phase, the HF rats had a significantly higher caloric intake compared to chow-fed animals (**Figure 1B**) (F (1, 46) = 93.60, *p* < 0.0001). As the rats reached adulthood, nearing the end of Phase I, the HF-HF rats weighed significantly more than the C-C rats (**Figure 1C**) (F (1, 46) = 21.13, *p* < 0.0001), signifying that the high-fat fed rats successfully underwent diet-induced obesity.

In Phase II, the rats either maintained their assigned diets (C-C, HF-HF) or were switched from HF to *ad libitum* chow (HF-C) or 60% chow caloric restriction (HF-CR). The HF-C group significantly increased their caloric intake from the onset of Phase II through the end of the experiment (p<0.03), indicating a hunger state, and suggesting a new set-point for hunger/satiety. The HF-CR group consumed a restricted stable diet throughout Phase II (**Figure 1C**). At the end of the experiment, there was no significant caloric difference in intake between C-C and HF-C (p=0.89) (Main effect of diet on intake in Phase II: F (3, 44) = 169.4, *p* < 0.0001).

During Phase II, HF-HF continued to gain weight, while C-C reached a plateau in weight gain. While HF-C showed a trend towards lower body weight, only HF-CR had a significant decrease in body weight (p<0.001) (**Figure 1B**). At the time of sacrifice, HF-CR weighed less significantly than the other groups (p<0.01), and there was no significant difference in body weight between the HF-HF and HF-C groups. HF-HF consistently weighed more than the C-C group throughout both phases of the experiment. Changing diets from HF to chow is considered a mild dietary manipulation, as there is a small, insignificant reduction in body weight, because the set point for hunger/satiety has been altered (Main effect of diet on body weight in Phase II: F (3, 44) = 8.212, *p <*0.001). These results show that consistent daily caloric restriction results in weight loss.

After establishing this DIO/CR model, we assessed explorative behaviour, to validate the model with other established obesity and weight loss models. The rats underwent two 5-minute explorative tasks, first the Open Field Test (OFT) followed by the Light-Dark Box (LDB) where their locomotion was recorded and analyzed. The results from these assays indicated that the CR exhibit a high level of explorative behaviour (**Figures 1D, E**).

In the OFT, HF-CR rats demonstrated more rearing (F (3, 41) = 8.148, *p <*0.001) (**Figure 1D**) and in the LDB, HF-CR demonstrated a higher count of crossing between chambers (F (3, 40) = 7.911, *p*<0.001) (**Figure 1E**) compared to the other groups.

To determine the dynamics of the energy-balance related transcripts that are commonly expressed in the ARC, we next checked the gene expression of *AgRP* and *Pomc*. *AgRP* mRNA expression was downregulated in HF-HF (p < 0.05, compared to C-C), incrementally increased in HF-C and significantly upregulated in the HF-CR group (p <0.0001) (ANOVA, F (3, 63) = 8.1, *p* < 0.0001, **Figure 1F**). *Pomc*, which is cleaved to α-MSH, a satiety hormone that counterbalances the effects of AgRP, was non-significantly upregulated in obese rats but was significantly downregulated in the HF-CR group (compared to HF-HF, p< 0.001) (ANOVA: F (3, 50) = 5.4, *p <*0.01; **Figure 1G**).

### 3.2 Caloric restriction reduces body weight, alters expression of classic hypothalamic energy-balance genes in DIO-male rats

**Figure 2:**
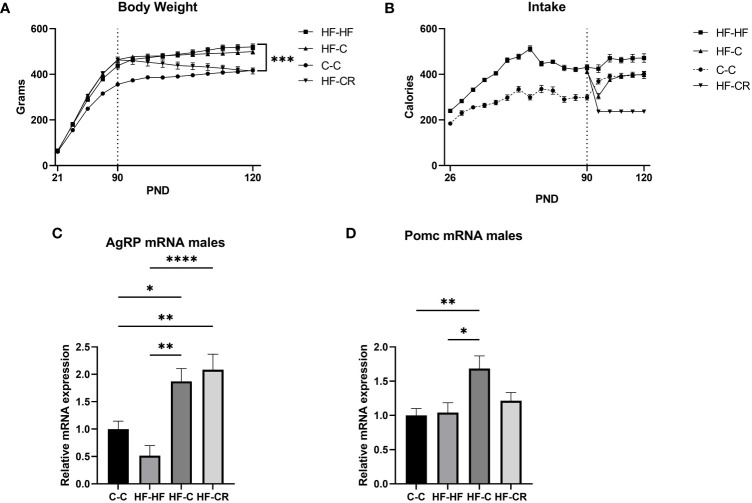
Caloric restriction results in reduction of body weight and changes in classic-energy balanced gene expression in the ARC of diet-induced obese male rats. **(A)** The average body weight (grams) throughout the duration of the experiment. In Phase I, rats were weighed every 5 days and weighed daily in Phase II. **(C)** Average caloric intake (kCal) throughout the duration of the experiment, measured by the weight difference of food remaining in the cages over 5 days during Phase I and daily in Phase II (Chow: 1 g = 3.1 kCal; HFD: 1 g = 5.24 kCal). Gene expression of the ARC was measured using RT-qPCR with primers designed for **(C)** AgRP and **(D)** Pomc. Hprt expression was used as the standard gene to normalize all results. Relative gene expression in the C-C group was set to 1. Data are presented as mean ± SEM, and significant effects between groups are indicated as * 0.01 < P < 0.05, **0.001 < P < 0.01, **** P < 0.0001.

While the results from the female rats were compelling, we wanted to check the physiological and transcriptional dynamics in males too (**Figure 2**). In Phase I of the experiment, male Wistar rats were raised on either chow or HFD and the HF rats had a significantly higher caloric intake compared to chow-fed animals (**Figure 2B**) (F (1, 46) = 176.9, *p* < 0.0001). As the rats reached adulthood, nearing the end of Phase I, the HF-HF rats weighed significantly more than the C-C rats (**Figure 2A**) (F (1, 46) = 85.51, *p* < 0.0001), signifying that the high-fat fed rats successfully underwent diet-induced obesity.

In Phase II, the rats either maintained their assigned diets (C-C, HF-HF) or were switched from HF to *ad libitum* chow (HF-C) or 40% chow caloric restriction (HF-CR). Upon the start of Phase II, the HF-HF, HF-C and C-C each slightly increased their caloric intake, and quickly reached a plateau through the end of the experiment. The HF-CR group consumed a restricted stable diet throughout Phase II (**Figure 1B**). At the end of the experiment, there was no significant caloric difference in intake between C-C and HF-C. (Main effect of diet on intake in Phase II: F (3, 44) = 77.01, *p* < 0.0001).

During Phase II, HF-HF, HF-C and C-C continued to gain weight. HF-CR had a significant decrease in body weight (p<0.001) (**Figure 2B**). At the time of sacrifice, HF-CR weighed significantly less than HF-HF and HF-C (p < 0.001), but there was no significant difference in body weight between the C-C and HF-CR. HF-HF consistently weighed more than the C-C group throughout both phases of the experiment. (Main effect of diet on body weight in Phase II: F (3, 44) = 23.54, *p* < 0.0001). These results show that, as in females, consistent daily caloric restriction results in weight loss in males.

We next checked the gene expression of *AgRP* and *Pomc*. *AgRP* mRNA expression was not significantly downregulated in HF-HF (p = 0.3, compared to C-C), but was upregulated in HF-C (p=0.0406) and HF-CR group (p <0.01) compared to C-C (ANOVA, F (3, 33) = 11.83, *p* < 0.0001, **Figure 2C**). There was no difference in *Pomc* expression between C-C and HF-HF (p =0.99), but HF-C was upregulated (p <0.01) compared to C-C. Further, the expression of *Pomc* was not significantly changed in HF-CR compared to C-C (p=0.6544) or HF-HF (P=0.8262) (ANOVA: F (3, 39) = 5.183, *p <*0.01; **Figure 2D**).

### 3.3 Enhanced H3K9me2 binding on the promoter of AgRP in female obese rats is reversed through diet

The significant differences in AgRP expression between C-C and HF-HF female rats were very compelling to our research and they were followed-up. As an orexigenic peptide, high levels of AgRP may indicate drive to feed and low energy expenditure hunger, while low levels would suggest satiation, or increased satiation signaling. However, appetite, or appetite-signaling is dysfunctional in conditions of undernutrition (i.e. starvation or pathology) or over nutrition (i.e. obesity) (64). Our model, and many others indicate that in a chronic obesogenic environment, rats continue to eat, even with a very low satiation signal.

**Figure 3:**
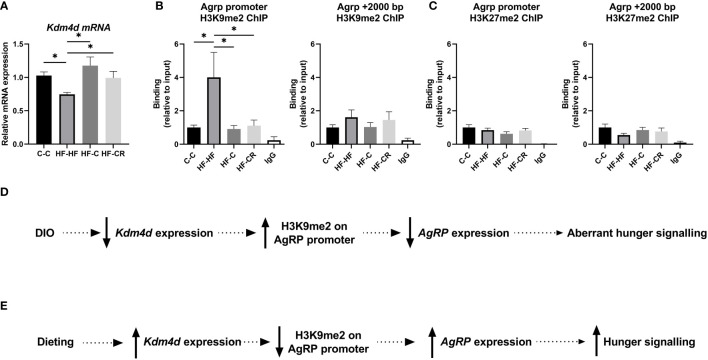
**(A)** Kdm4d gene expression of the ARC was measured using RT-qPCR. Hprt expression was used as the standard gene to normalize all results. Relative gene expression in the C-C group was set to 1. **(B)** To check the histone methylation status of the target region, ChIP-qPCR was performed with anti-H3K9me2 and primers aligned to the promoter of AgRP and a control region 2000 base pairs downstream from the AgRP promoter. **(C)** To check the specificity of the histone modification location at the AgRP promoter, the enrichment of H3K27me2 was assesses using ChIP-qPCR, at the location of AgRP promoter and 2000 bp downstream. **(D)** Schematic presentation of proposed chromatin repressive pathway in obesity. In diet-induced obese rats, Kdm4d expression is downregulated leading to less demethylation of residue H3K9 at the AgRP promoter. The high abundance of repressive histone marker H3K9me2, leads to a downregulation in AgRP expression. This change in hypothalamic energy balance signaling partially effects the aberrant hunger signals in obesity. **(E)** However, this signaling is reversed through dieting. In dieting, Kdm4d expression is upregulated leading to more demethylation of residue H3K9 at the AgRP promoter. In the absence of a strong repressive marker, AgRP expression is upregulated, and rats exhibit a rebound, normal hunger signal. Data are presented as mean ± SEM. Significant effect between groups is indicated by *0.01 < P < 0.05 using ANOVA test with Tukey for multiple comparisons. For the ChIP experiments, the IgG was significantly different (P < 0.05) than the other groups, but not indicated for graphic simplicity.

To understand how the methylation status of lysine 9 histone 3 effects energy balance, we focused on the role of the enzyme KDM4D, as it specifically demethylates H3K9 and has been shown to be involved in obesity (43–48). We found that DIO-rats expressed a downregulation of *Kdm4d* mRNA (p<0.05) and importantly, obese rats that underwent a diet change, either caloric restriction on chow or simply a diet change from exclusively feeding on HFD to *ad libitum* chow, displayed a rebound upregulation of *Kdm4d* mRNA expression to chow-*Kdm4d* levels. (F (3, 45) = 3.28, *p*= <0.05; **Figure 3A**). We were interested to explore if and how Kdm4d regulates this behaviour. To this end, we used chromatin immunoprecipitation to check the levels of H3K9me2 around the promoter of AgRP. Compared to C-C, HF-HF rats had significantly more H3K9me2 at the AgRP promoter (p<0.05) (**Figure 3B**). The groups that changed diets, HF-C and HF-CR, had a lowered enrichment of H3K9me2, compared to the HF-HF group (HF-HF vs HF-C: p< 0.05; HF-HF vs HF-CR: p< 0.05) (ANOVA: F (4, 47) = 3.7, *p <*0.05; **Figure 3B**). These change in diet groups returned to baseline of C-C enrichment of H3K9me2. A region 2000 base pairs downstream to the AgRP promoter in the same samples was used as a control region against the chromatin architecture of the promoter and found no significant differences of H3K9me2 enrichment between the groups (F (3, 36) = 0.7, *p* = 0.55; **Figure 3B**).

To strengthen the concept of spatially specific modulation of energy balance in obesity and dieting at H3K9, we assessed another transcriptional repressor, H3K27me2. There were no significant differences in H3K27me2 binding between the groups, either at the *AgRP* promoter (F (3, 37) = 1.2, *p* = 0.33; **Figure 3C**) nor downstream 2K base-pairs from the promoter (F (3, 38) = 1.0, *p* = 0.21; **Figure 3C**).

Together, these results indicated that in DIO (**Figure 3D**), downregulation of *Kdm4d* mRNA correlated with less demethylation of H3K9 on the *AgRP* promoter, leading to a repression of *AgRP* expression. This aberrant hunger signaling is in accordance with other irregular signaling that is found in the framework of obesity. Remarkably, we found that dieting (**Figure 3E**) removed the chromatin repressor modification *via* upregulated *Kdm4d* expression, correlating with high demethylation of H3K9 at the promoter of AgRP. In dieting rats, the hunger signal is restored due to demethylation of H3K9me2.

### 3.4 Pharmacological inhibition of KDM4D inhibits feeding

After establishing the correlative role of KDM4D modulation over AgRP, we next wanted to show that exogenous inhibition of Kdm4d could modulate the expression of *AgRP*. With the hypothesis that blocking the action of KDM4D in the ARC would decrease *AgRP* expression and in turn reduce feeding in rats, JIB-04 was used as a pharmacological KDM4D inhibitor to cross the BBB and affect *AgRP* expression in the ARC.

**Figure 4:**
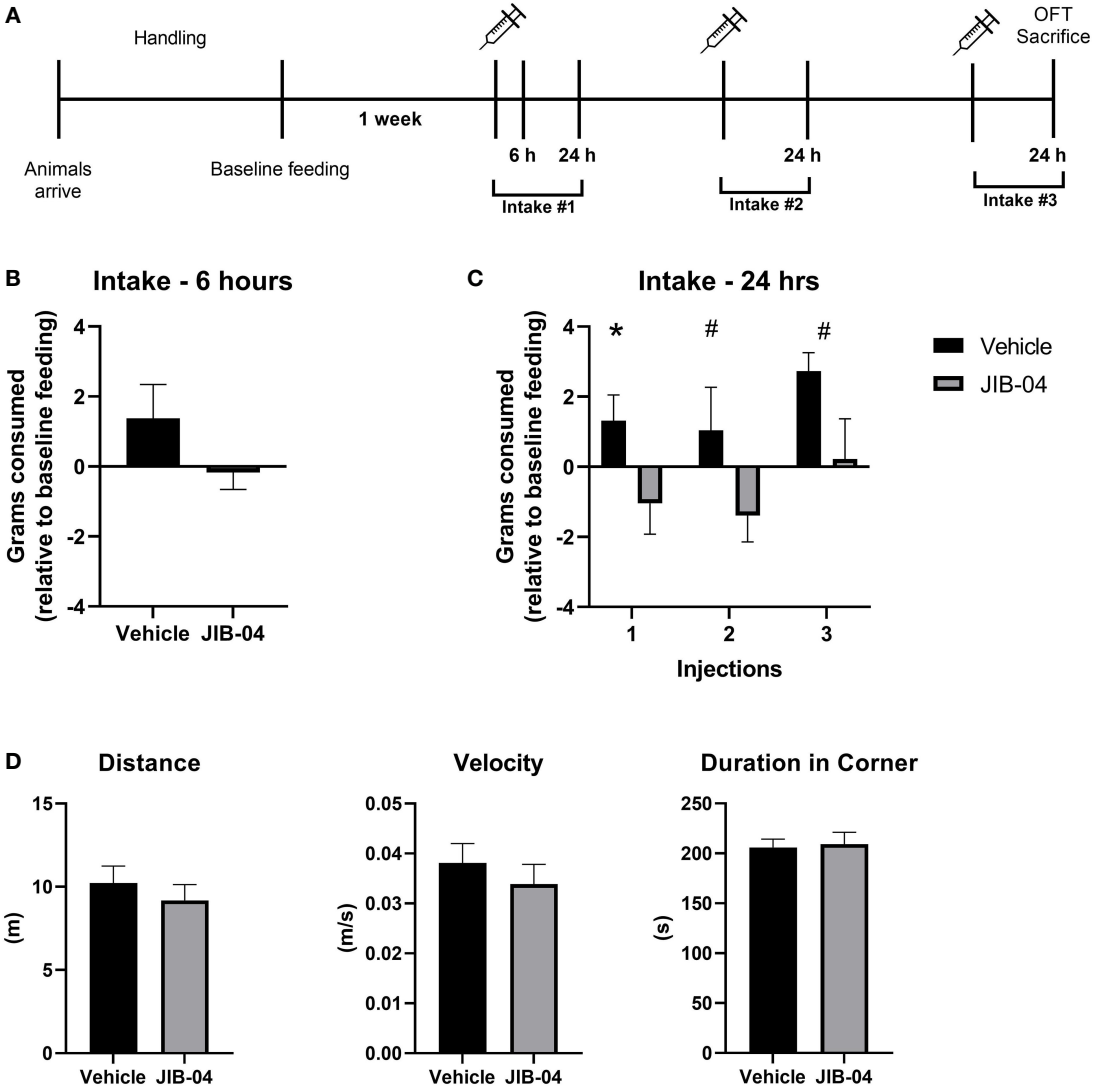
JIB-04 administration inhibits feeding behavior. **(A)** Experimental timeline. Naïve adult female Wistar rats underwent a period of acclimation and handling. Rats were housed individually for 24-hours to collect baseline feeding data. The following week, rats were injected (IP) with JIB-04 or vehicle solution, every-other day for a total of three treatments. Food intake for 24-hours post administration was compared to the baseline feeding measurement of each specific rat. 6 hours after the first injection, intake was measured in one cohort of rats. 24-hours after the final administration, rats were assessed in a standard Open Field Test and then sacrificed. **(B)** Food intake (grams of chow) 6-hours after first injection. The intake of individual rats was compared to their individual baseline intake. The intake of individual rats was compared to their individual baseline intake. n=5 per group. **(C)** Food intake (grams of chow) was measured 24-hours after each injection (1–3). The intake of individual rats was compared to their individual baseline intake. n= 13 per group. **(D)** At the end of the experiment, rats were exposed to the Open Field test (OFT). The parameter of *distance* (meter travelled) and *velocity* (m/s) are measurements of locomotion, and *duration in corner* measures that the time spend in any of the 4 corners, compared to the center of the arena. n= 13 per group. Data are presented as mean ± SEM, and significant effects between groups are indicated as # P < 0.1, P *<0.05 using an unpaired t-test.

The baseline feeding of naïve adult female Wistar rats was assessed prior to the onset of the experiment. Each rat was injected (IP) with either KDM4D-inhibitor JIB-04 (20 mg/kg), or vehicle solution three times. Food intake was measured 24-hours after each drug administration. In the first cohort of rats used in the experiment, feeding was also assessed 6 hours after the first injection (**Figure 4A**), to get a sense regarding the feeding pattern shortly after drug administration. Intake was normalized to individual feeding (within subject). After 6 hours, there was a (non-significant) pattern of feeding inhibition in JIB-04-treated rats compared to vehicle (t (12) = 1.4, *p* = 0.18) (**Figure 4B**). After 24 hours, rats that were administered JIB-04 consumed fewer grams (approaching significance) of chow compared to controls after each injection (injection 1: t (25) = 2.04, *p*=0.05; injection 2: t (25) = 1.78, *p* =0.08; injection 3: t (25) = 1.86, *p* = 0.07) (**Figures 4C**).

24-hours after the 3rd drug administration, the rats were subjected to the OFT to check general toxicity of the injected drug by measuring anxiety-like behaviours and locomotion. There were no significant differences found between JIB-04 and vehicle- injected rats in any of the parameters measured in the test (**Figure 4D**).

### 3.5 Histone methylation at H3K9 of the AgRP promoter alters satiation signaling

**Figure 5:**
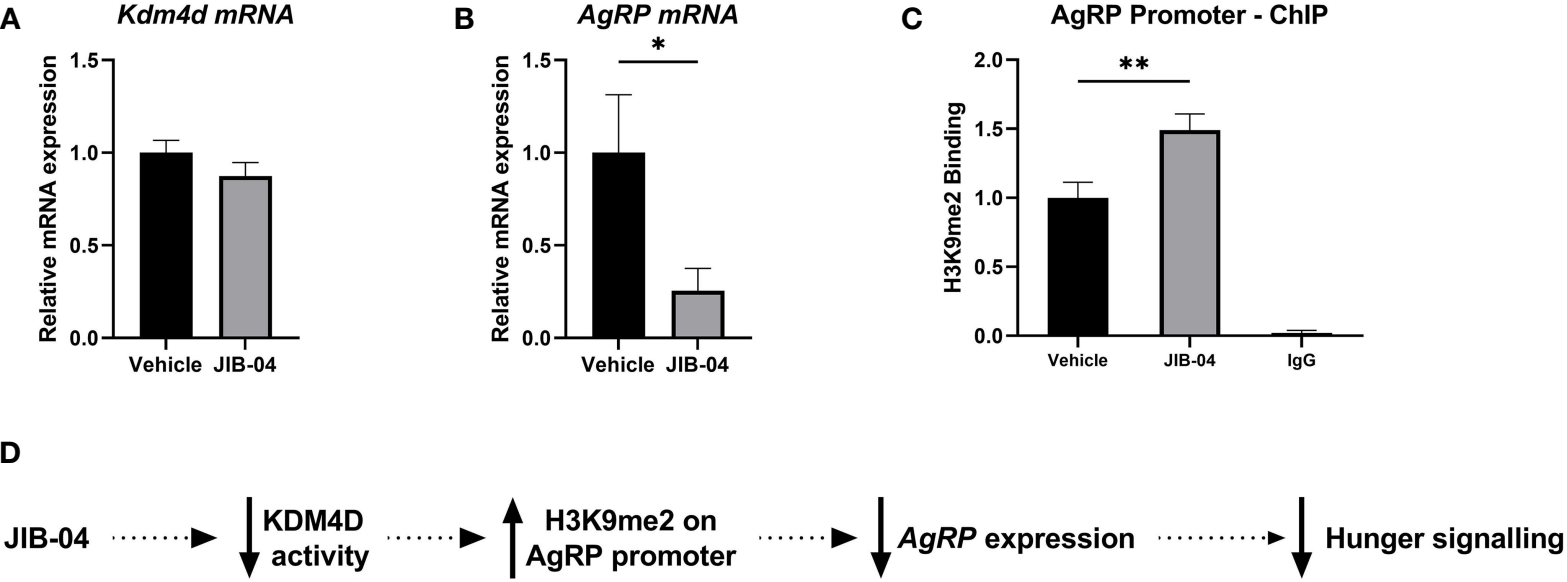
Histone methylation at H3K9 promotes satiation signaling and decreased feeding **(A, B)** Specific gene expression of the ARC was measured using RT-qPCR with primers designed for **(A)** *Kdm4d* and **(B)** *AgRP*. **(C)** ChIP-qPCR was performed with anti-H3K9me2 and primers aligned to the promoter of AgRP to assess histone enrichment. **(D)** Schematic presentation of proposed mechanism of de/methylation of histone tail, regulating AgRP expression. In JIB-04 treated rats, the activity of KDM4D is inhibited, cementing the di-methylation status of H3K9 on the AgRP promoter. This in turn represses *AgRP* expression, inhibiting the hunger signaling and decreasing feeding. Data are presented as mean ± SEM, and significant effects between groups are indicated as * 0.01 < P < 0.05, **0.001 < P < 0.01.

We used JIB-04 as a molecular inhibitor to block the activity of KDM4D. There was no significant difference in *Kdm4d* mRNA expression (t (23) = 1.2, *p* = 0.24) between the treatment groups (**Figure 5A**). However, as a result of KDM4D inhibition, *AgRP* expression was significantly downregulated in the JIB-04 treated group (t (23) = 2.4, *p* < 0.05) (**Figure 5B**). To check if the level of H3K9me2 on the promoter of AgRP was changed, we performed ChIP with H3K9me2 antibody. There was significantly more H3K9me2 enrichment at the AgRP promoter in the JIB-04 group, compared to the vehicle (t (13) = 3; *p <*0.001) (**Figure 5C**).

Together, these results indicate that when methylation at H3K9 is anchored on the promoter of AgRP, *AgRP* expression is in fact inhibited and there is a decrease in hunger signaling in the ARC, leading to lower food intake (**Figure 5D**).

## 4 Discussion

As the prevalence of obesity and associated metabolic disorders continues to rise at a drastic rate, it is critical to continue developing therapeutic interventions for weight loss (3). Obesity is a multifactorial, progressive metabolic disease in which epigenetics impact its diverse etiology and potential treatment pathways. Epigenetic modifications are dynamic, and because these modifications can be reversible (50, 51) they are attractive targets for designing new treatments for overeating and obesity (9, 14, 65, 66). We focused on a mechanism by which methylation of the cytoplasmic tail of histone 3 at lysine 9 regulates hypothalamic energy balance signaling. In this work, we found that caloric restriction reverses the activity of KDM4D by actively demethylating the histone tail of the AgRP promoter in obese rats, thus reducing their food intake.

We were interested in uncovering specific epigenetic mechanisms governing weight-loss in obese animals. Previously in our lab, we found that DIO male rats exhibited hypermethylation along the CpG sites on the *Pomc* promoter in the ARC, impairing Sp1 transcription factor binding (32). DIO female rats also showed similar malprogramming on the *Pomc* promoter, and these epigenetic modifications were propagated to their offspring, even in the absence of an obesogenic environment after weaning (29). The offspring maintained an obesogenic phenotype, with higher body weight and poor results in a *high-fat diet challenge* later in life (29). Interestingly, while virgin DIO-female rats expressed hypermethylation on the *Pomc* promoter, this hypermethylation was not present in dams after pregnancy and lactation, suggesting that a drastic energy expenditure could lead to a reversal of these epigenetic modifications (29).

Here, we utilized a model of dieting after diet-induced obesity, in order to compare the transcriptional expression and chromatin architecture in the ARC between standard diet-fed rats (C-C), DIO rats (HF-HF) and two levels of dieting, mild (HF-C) and strict (HF-CR). The HF-C group was designed both as a control for HF-CR, in which diet was changed (HFD to standard chow), but also to represent a moderate diet, changing from a predominantly fat-based diet to a balanced diet, however, without restriction. The HF-CR group was designed as an extreme diet, with a 60% reduction in daily caloric intake. This calculation was derived from previous work in our lab (unpublished) and other models (59, 67–71) in which rodents steadily lose weight but do not become malnourished (72).

Caloric restriction (CR) has been traditionally used as a paradigm for investigating mechanisms of aging and longevity (59, 68, 69). Studying various models of CR, including the scheduling of feeding times has been popular in recent years as it has been shown to improve cardiac function (63), blood glucose levels (73) and overall inflammation (59, 67). Specific epigenetic mechanisms have been pinpointed to CR promoting lifespan (69, 71). For example, in response to CR in rats, SIRT1 was upregulated, inhibiting stress-induced apoptosis in cells by removing the acetylation on a DNA repair protein, Ku70 (70). In our work, we used this established paradigm to specifically induce significant weight loss in obese rats, with the hypothesis that epigenetic mechanisms guide the phenotypic changes.

Body weight of each treatment group maintained the expected trajectory, with HF-CR losing weight during dieting phase. HF-C rats had a slight drop in weight immediately after diet change, but then plateaued, as they increased their daily caloric intake indicating hunger and adjustment to the new set-point for hunger/satiation. The groups with maintained diets (C-C and HF-HF) had a plateau body weight throughout adulthood. In both diagnostic behavioural tests, HF-CR rats were highly active – performing more rearing (OFT) and crossing more lines (LDB), compared to the other groups, which may represent exploration for food, or hunger drive (74, 75). CR-rats have been found to have mild reduction in anxiety-like behaviours, compared to ad libitum-fed rats in the elevated plus maze and in the OFT (76, 77). Further, Levay et al. (76) showed a caloric restriction- dose-dependency effect of anxiolytic behaviour in the OFT; less anxiolytic behaviour in 50% caloric restriction compared to 25% caloric restriction. The literature is mixed regarding anxiolytic behaviours in HFD-fed rats; some studies have found that in female rats there are no differences between chow- or high-fat-fed rodents in OFT behaviours (78, 79), but other have found that high fat diet led to more anxiolytic behaviour (80). Taken together, the phenotype presented in this study is comparable to previous models of DIO and caloric restriction and therefore a valid model to work with.

After developing our model of caloric restriction after diet induced obesity, we were interested in assessing the chromatin architecture in the ARC as a specific mechanism by which epigenetics modulate weight loss in obesity. H3K9 methylation became a prospective marker for further investigation because of its systemic (43–48) and neural (47, 49) roles in obesity and its strong repressor function of chromatin condensation (42). We were interested in understanding how H3K9me governs changes in the ARC structuring through weight gain and weight loss. Chromatin accessibility can be altered by proteins in the histone methyl transferase (HMT) family or histone demethylases (HDM) family, which are recruited under various conditions (81) to add or remove methyl groups (82). These modifications not only affect the chromatin structure by merely being there, they also recruit proteins and complexes with specific enzymatic activity to alter transcription (83, 84). KDM4D (lysine demethylase 4D) is a HDM that specifically demethylates H3K9me2 and H3K9me3 (45, 52, 53).

We began our molecular investigation by quantifying the mRNA expression of *Kdm4d* in the ARC. We found a significant downregulation of *Kdm4d* transcription in HF-HF compared to chow- fed rats. Interestingly, there was an upregulation recovery, back to baseline of *Kdm4d* expression in the dieting groups. Quantifying this profound transcriptional rebound of a gene whose protein is responsible for demethylation of a strong repressive marker characterized in obesity was compelling, so we next decided to assess the expression of other transcripts involved with energy balance, to understand if and how KDM4D may modulates their expression. We note that a histological analysis would have potentially demonstrated that *Kdm4d* is expressed in *AgRP* neurons. However, both available antibodies to *Kdm4d* were found to be unspecific in both Western blots and ChIP analyses.

The mRNA expression of the classical energy-balance genes in the ARC was assessed. We found that there was no significant difference in *Pomc* expression between C-C and HF-HF, but a significant downregulation in expression in the dieting groups, compared to HH-HF, strongest in the HF-CR group. Although POMC is an anorexigenic peptide that tends to counterbalance AgRP, the insignificant difference in *Pomc* expression between C-C and HF-HF fits with previous findings that there is abnormal transcription of *Pomc* mRNA in the ARC in an obesogenic environment (29, 49). However, the levels of *Pomc* transcription were reduced, as expected, in the more extreme condition of caloric deficit, as a survival, “drive-to-eat” signal. The aberrant signaling of POMC due to chronic HFD was repaired through dieting and caloric restriction.

We found that DIO led to downregulation of orexigenic neuropeptide *AgRP* expression. Even though the *AgRP* transcription was low, in this obesogenic environment with an abundance of high-fat food available, rats continued to eat and maintain an energy balance surplus. It has been well established that high-fat diet is a rewarding food for rodents, triggering hedonic overfeeding (64, 85) and feeding *post satiation* (86). Further, obesity leads to aberrant signaling in the ARC (29, 32, 87–90) and other hypothalamic structures (22, 26, 47, 90, 91), so it was not surprising to quantify low *AgRP* expression whilst rats continue to over-consume. Even with excess energy stores and elevated levels of circulating leptin and insulin, DIO rodents consume more calories than their lean counterparts (32). A recent study showed that transcriptional repression of *AgRP* leads to a sedentary phenotype (36), so repression of *AgRP* mRNA in obese rats may not only correspond with overfeeding, but also with reduced energy expenditure, together, perpetuating the obese phenotype. Here we found that in the dieting groups, the expression of *AgRP* was positively correlated with the stringency of diet; *AgRP* was slightly upregulated in the mild dieting condition, and strongly upregulated in the calorically restricted group. Obese rats that undergo dieting exhibited a rebound effect on *AgRP* transcription.

In our previous work, we explored the modulation of H3K9me2 and H3K9me3 over the *Pomc* promoter (92), so our next step was to understand if and how histone accessibility modulated by H3K9me2 does in fact regulate AgRP transcription. We performed ChIP with H3K9me2 antibody and found increased binding to the AgRP promoter in HF-HF compared to C-C rats, and critically, this high binding level returned to baseline in both dieting groups. As proof of concept that this modification was specific to the promoter of *AgRP*, we quantified the enrichment of H3K9 methylation in a genomic area 2000 base-pairs downstream, and we found no methylation differences between the groups. To test the specificity of H3K9me2 in the reversal of *AgRP* transcription in obesity, we assessed an additional chromatin repressor (H3K27me2) using the same samples and found no difference in this modification between groups at the location of *AgRP* promoter nor 2000 base pairs downstream. As the hypothalamus is a complex neuronal network with a remarkable range of cell types, and there is a delicate balance between satiety and hunger signals regulated through counter expression of anorexigenic and orexigenic neuropeptides, it is important to state that it is likely that other neuropeptides in this area are regulated by the level of methylation on K3H9 and hence will be affected by JIB-04. Thus, the changes in appetite may be caused by other genes in addition to changes in histone methylation on the *AgRP* promoter.

Uncovering similar recovery patterns caused by dieting after chronic HFD in both i) AgRP transcription and ii) H3K9me2 binding to the AgRP promoter led us to hypothesize that diet modulates *Kdm4d* expression, which in turn affects histone methylation around the *AgRP* promoter and *AgRP* transcription, effecting feeding. Therefore, the next step was to show that KDM4D is necessary and sufficient to modulate the expression of *AgRP* in the ARC.

To elucidate the functional role of KDM4D in reversing obesity *via* dieting, a pharmacological agent JIB-04 was used to inhibit KDM4D *in vivo*. JIB-04, first synthesized in 2012, is a pan-selective inhibitor of many of the proteins in the KDM family, with a high selectivity for Kdm4d (55). Because KDMs are highly expressed in glioblastoma (57) and hepatocellular carcinoma tumors (56), potent KDM inhibitors have been targeted as therapeutic agents for these cancers. JIB-04 has been used both *in vitro* to inhibit cancer cell activity and *in vivo* to inhibit different types of cancer growths (55–57) and increase survival rates in mice (55). Unlike other KDM inhibitors, JIB-04 is the only known agent to function *in vivo* and successfully pass the blood-brain barrier (57). Through inhibiting the demethylation of H3K9me2, JIB-04 treated rats expressed a downregulation of AgRP and decreased food intake over a 24-hour period.

In this experiment, naïve rats were handled to minimize stress prior to the experiment. Before food intake assessment, rats were fasted overnight, a protocol used to ensure a uniform level of hunger between all animals (93). For 24 hours after drug administration, rats were housed individually to collect individual feeding data.

The dosage of 20 mg/kg of JIB-04 used in this experiment was the lowest dose found in the literature to cross the BBB and have a clinically relevant amount in the brain (57). It was three times lower than doses used in cancer treatments. At high doses (100-110 mg/kg), Banelli et al. (57) reported adverse neurological side effects and potential interactions with other cancer-treatments. The rats in the present experiment were assessed in a standard Open Field Test to check for toxicity effects, including changes in mobility or anxiety-like behaviors. There was no difference in OFT performance, indicating that the inhibition of feeding was in fact due to changes in the satiation/hungry pathways, rather than the locomotion, motivation, or anxiety- pathways. Further, at this dosage, JIB-04 is not expected to induce adverse reactions in rats (57).

After each drug administration session, Kdm4d-inhibited rats fed less than the controls. It should be noted that there is a reduction in the effect of the drug with multiple injections that can be due to a feedback or a saturation effect. We did not conduct a longer injection protocol because the first injection was effective and thus answered our biological question. Gene expression of *AgRP* and *Kdm4d* was quantified after sacrifice and supported the hypothesis. Inhibiting KDM4D with JIB-04 did in fact downregulate *AgRP* expression in the ARC, while no differences were found in Kdm4d expression. Modifying the methylation status of H3K9 with JIB-04 will be expected to have effects on histone methylation outside of the ARC, such as altering adipogenesis (94, 95). These changes may contribute or even indirectly drive the observed changes in food intake. It should be emphasized that although affecting one histone modification, as described in this manuscript, alters the expression to a level that affected the satiety of the rats, histone modifications work in concert and one modification may not characterize the whole regulatory effect, in which other epigenetic marks might be involved.

According to our knowledge, this is the first time that this compound, JIB-04, has been used to successfully inhibit feeding, without adverse side effects. Potential drug-associated illnesses (which to our knowledge have never been checked), as modelled by the conditioned taste aversion test, should be considered in future studies. In our study, the compound was administered intraperitoneally, as IP-injection is routinely performed in our lab, however, the literature states (55, 57) that neither administration of JIB-04 by intraperitoneal injection or by oral gavage has negative side effects in a rodent model, which increases the translational potential for therapeutic development.

This work supports the idea that epigenetic modifications are plastic and reversible and can moderate changes in obesity and metabolic disorder. We propose that the action of KDM4D through the demethylation of H3K9 is both necessary and sufficient in maintaining a stable epigenetic landscape on the *AgRP* promoter in the hypothalamus. This may offer a target for developing new treatments for overeating and obesity.

## Data availability statement

The raw data supporting the conclusions of this article will be made available by the authors, without undue reservation.

## Ethics statement

The animal study was reviewed and approved by Bar-Ilan University Animals Care and Use Committee.

## Author contributions

KR, AW, and NM designed research. KR and TK performed research. KR, TK, and AM analyzed data. KR, AW and NM wrote the paper. All authors contributed to the article and approved the submitted version.
